# Evolution of H5N1 Cross‐Species Transmission: Adaptive Mutations Driving Avian‐to‐Human Infection

**DOI:** 10.1002/ggn2.202500051

**Published:** 2026-01-11

**Authors:** Wenxin Man, Lin Du, Ying Liu, Zehan Pang, Hongyan Zhu, Bixia Hong, Zhichao Xu, Huahao Fan

**Affiliations:** ^1^ College of Life Science Northeast Forestry University Harbin China; ^2^ State Key Laboratory of Synthetic Biology Tianjin University Tianjin China; ^3^ College of Life Science and Technology Beijing University of Chemical Technology Beijing China; ^4^ College of Biology and Brewing Engineering Taishan University Taian China; ^5^ School of Basic Medical Science State Key Laboratory of Respiratory Disease Guangzhou Medical University Guangzhou China

**Keywords:** adaptive mutations, cross‐species transmission, evolution, H5N1, influenza epidemic

## Abstract

First detected in poultry in China in 1996, the H5N1 avian influenza virus has evolved into a significant global public health hazard, primarily owing to its high pathogenicity and potential for interspecies transmission. While primarily affecting avian species, H5N1 has repeatedly breached species barriers, infecting mammals including humans, minks, seals, and cattle. This review synthesizes current knowledge on the molecular mechanisms underpinning H5N1's host adaptation, focusing on key mutations in viral proteins‐such as hemagglutinin (HA), neuraminidase (NA), and polymerase subunits (PB2)‐which boost binding affinity to human‐type receptors, increase replicative efficiency in mammalian cells, and facilitate immune evasion. Critical mutations, including HA‐Q226L, HA‐T199I, PB2‐E627K, and NA‐H274Y, are discussed in detail, highlighting their roles in altering receptor specificity, promoting antiviral resistance, and expanding viral tropism. The paper also outlines epidemiological trends, global dissemination patterns driven by migratory birds and trade, and current strategies for prevention and control, including antiviral therapeutics and vaccine development. Ultimately, this comprehensive analysis underscores the urgent need for continued surveillance, broad‐spectrum countermeasures, and international collaboration to reduce the pandemic risk posed by H5N1.

## Graphical abstract

This review synthesizes current knowledge on the molecular mechanisms underpinning H5N1's host adaptation, focusing on key mutations in viral proteins. Critical mutations are discussed in detail, highlighting their roles in altering receptor specificity, promoting antiviral resistance, and expanding viral tropism. The review underscores the urgent need for continued surveillance, broad‐spectrum countermeasures, and international collaboration to reduce the pandemic risk posed by H5N1.

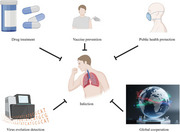

## 1 Overview of the H5N1 Avian Influenza Epidemic

**FIGURE 1:**
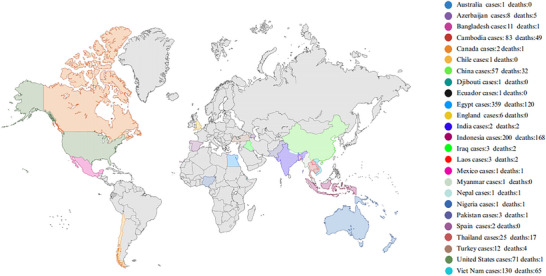
Distribution map of the total confirmed human cases of avian influenza A (H5N1) reported to the WHO between 2003 and 2025. Different colors indicate the number of confirmed cases (cases) and deaths (deaths) reported by various countries. Readers can find the corresponding relationship between specific countries and the data in the right‐hand legend.

**FIGURE 2:**
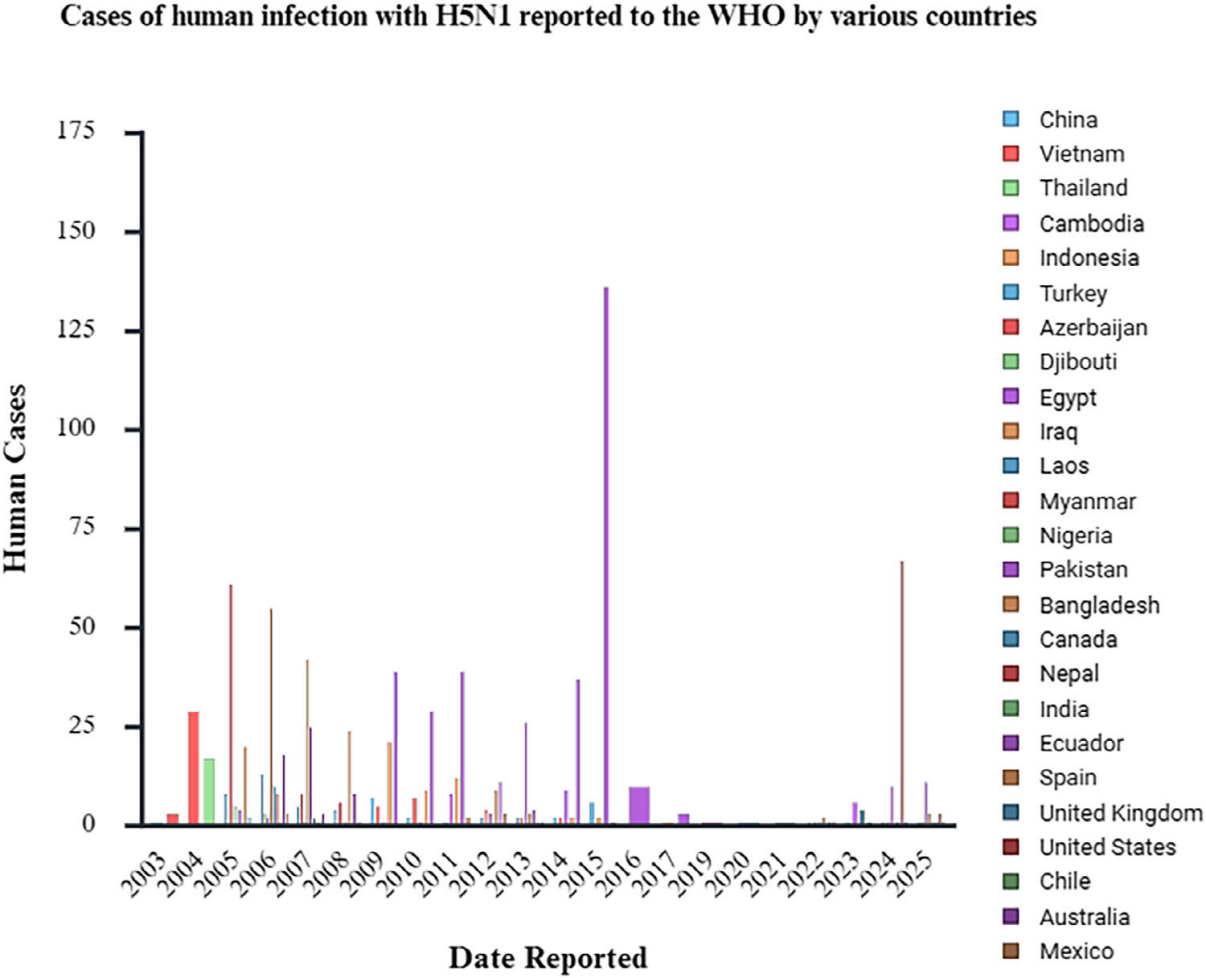
Human H5N1 Infection Cases Reported to the WHO by Various Countries. The bar chart shows the distribution of human avian influenza cases reported to the WHO by various countries/regions from 2003 to 2025. The bar columns of different colors correspond to the respective countries/regions. The vertical axis denotes the number of human cases, while the horizontal axis indicates the reported years, illustrating the number of cases each country reports annually.

H5N1 is a highly pathogenic strain of avian influenza virus that was first detected in poultry in Guangdong Province, China, back in 1996. The subsequent year, 1997, marked the first documented human infections in Hong Kong, China, prompting significant concern regarding the virus's potential for interspecies transmission, particularly from avian hosts to humans, despite the absence of sustained human‐to‐human transmission at that time [1, 2, 3]. Presently, numerous countries and regions have reported cases and fatalities associated with H5N1, underscoring its widespread dissemination (Figure 1). Between 2003 and 1 July 2025, the World Health Organization (WHO) documented 985 human infections with the highly pathogenic H5N1 avian influenza across 25 countries or regions, leading to 473 fatalities (Figure 2), thereby highlighting H5N1 as a significant global public health concern [4].

The virus predominantly affects waterfowl and domestic poultry and is prevalent among wild and domestic bird populations in Asia. Sporadic and transient instances of avian‐to‐human transmission persist, with most human infections strongly associated with exposure to infected poultry [1, 5, 6]. The genetic variation and mutational potential of the virus in avian hosts have led to the development of multiple genotypes and sub‐lineages, thereby enhancing the likelihood of viral adaptation to diverse hosts and presenting significant challenges to epidemiological control efforts [7, 8]. Furthermore, various transmission pathways, including migratory wild birds, poultry trade, and transportation networks, have facilitated the global dissemination of the H5N1 virus [9, 10]. Bayesian inference analyses, utilizing subtype gene sequences from Asia and Russia, have demonstrated that H5N1 propagates along distinct avian migratory routes, with waterfowl following the Central Asian migration corridor contributing crucially to the virus's long‐distance transmission [9, 11, 12]. For example, the extensive outbreak at Qinghai Lake in 2005 illustrated that migratory wild waterfowl, such as the bar‐headed goose, acted as a “bridge” for virus transmission, promoting the migration and prevalence of the virus to Europe and Africa [12, 13].

Since its initial detection in poultry in Guangdong, China, in 1996, the virus has undergone significant evolution, expanding from a localized area in Asia to multiple regions worldwide, and has consistently breached species barriers. It has infected a diverse array of animals, including geese, chickens, waterfowl, minks, seals, cattle, and sheep (Table 1), leading to an unparalleled number of outbreaks in wild avian species, backyard poultry, and birds in rural and farm settings. These events underscore the expanding host range of the virus and its ongoing pandemic threat. Notably, the 2.3.4.4b lineage, initially identified in North America in 2021 and subsequently detected in Antarctica in 2023, has emerged as the predominant driver of recent global outbreaks, including infections in novel mammalian hosts [14]. The transmission of the virus from North America to cattle, leading to subsequent human cases, alongside infections in sheep in Europe, underscores the extensive global dissemination of the H5N1 virus's 2.3.4.4b lineage [15, 16].

**TABLE 1:** Line of H5N1 Cross‐Species Transmission Events.

Year	Country/Region	Species	Event description	Significance
1996	China (Guangdong)	Goose (Poultry)	First detection of H5N1 virus, with HA gene similar to the 1997 Hong Kong strain [19]	Marks the first large‐scale outbreak of H5N1 in Asia, laying the groundwork for future cross‐species transmission
1997	China (Hong Kong)	Chicken (Poultry), Human	First human infection cases, high pathogenicity [2]	The H5N1 virus is first confirmed to have the potential to transmit directly from poultry to humans
2003–2008	Asia, Europe, the Middle East, and Africa	Wild Birds, Poultry	Virus spreads rapidly from Asia to many countries globally [20]	Completes the transition from localized outbreaks to the global spread, becoming a significant public health threat
2005	Global	Wild Waterfowl, Poultry, Human	Virus widely spreads in migratory birds and further transmits to poultry and humans [21]	Migratory birds become an important medium for cross‐continental transmission, increasing the global spread risk.
2022	Spain	Poultry, Mink	First report of H5N1 transmission from poultry to mink on a farm [22]	The virus first achieves cross‐species transmission in farmed animals, adapting to new host environments
2023	Argentina	Wild Birds, Seal	Outbreak of H5N1 in seals, mass death of elephant seals [23]	The virus first establishes sustained transmission in marine mammals, expanding its host range
2024	USA	Wild Birds, Cattle	Multiple outbreaks of H5N1 in cattle farms [24]	Efficient transmission in cattle, significant host adaptation, and potentially transmissible to humans
2025	USA	Wild Birds, Cattle, Human	Ongoing spread of the H5N1 virus in cattle, with the widespread 2.3.4.4b subtype [17]	Virus shows increased adaptability and cross‐species transmission potential
2025	Global	Poultry, Wild Birds, Cattle, Mink, Seal	2.3.4.4b subtype widely transmits into multiple mammal species [25]	Virus shows enhanced adaptability and cross‐species transmission potential

In contrast to other H5N1 clades, such as clades 2.2 and 2.3.2.1, the 2.3.4.4b lineage has developed a distinct set of adaptive mutations and transmission characteristics that are fundamental to its heightened transmissibility and infectivity in mammals. Within bovine hosts, this lineage demonstrates the ability to efficiently replicate in bovine respiratory epithelial cells, a capability that is largely absent in most other H5N1 lineages [17]. Regarding human hosts, the 2.3.4.4b lineage exhibits a markedly increased binding affinity for human‐type α‐2,6 sialic acid receptors and demonstrates enhanced replication adaptability in human respiratory cells. This distinguishes it from earlier lineages, which are predominantly restricted to avian hosts, infect humans only sporadically, and exhibit very low transmission efficiency [18].

This review systematically synthesizes key adaptive mutations and the underlying molecular mechanisms involved in the cross‐species transmission of the H5N1 avian influenza virus. It emphasizes the role of mutations in viral proteins such as HA, NA, and polymerase subunits (e.g., PB2) in facilitating host adaptation from avian to mammalian species, including humans. Additionally, the article integrates global epidemiological trends and transmission patterns, evaluates the current spread and pandemic potential of H5N1, and outlines comprehensive prevention and control strategies, encompassing antiviral therapeutics and vaccine development. By synthesizing these multidimensional perspectives, this review aims to provide a robust scientific foundation for future surveillance, drug design, and public health responses to H5N1.

## 2 Mutations in H5N1 and Their Impacts

The Influenza A virus subtype H5N1 is categorized within the genus Alphainfluenzavirus, belonging to the Orthomyxoviridae family, and is identified as a highly pathogenic avian influenza (HPAI) subtype [26]. The Influenza A virus genome consists of eight distinct negative‐sense single‐stranded RNA segments, each encapsulated as an individual ribonucleoprotein complex (RNP). These segments collectively encode the viral proteins PB2, PB1, PA, HA, NP, NA, M, and NS, facilitating efficient reassortment and adaptation to host organisms (Table 2) [27]. Virions exhibit spherical or pleomorphic morphologies, with diameters ranging from 80 to 120 nm, and possess an outer envelope. The core of the virion contains a helical RNP composed of the nucleocapsid protein (NP), three polymerase subunits (PB2, PB1, PA), and the single‐stranded negative‐sense segmented RNA genome. The envelope, a bilayered lipid membrane, is studded with the proton channel M2 and glycoprotein spikes‐HA and NA‐at an approximate molar ratio of 4:1 [26, 28]. HA, a key molecular determinant of viral dissemination, is essential for productive infection. This homotrimeric glycoprotein comprises a globular head domain and a membrane‐proximal stem domain [29]. The globular head domain of HA facilitates high‐affinity recognition and attachment to terminal sialic acid receptors present on host‐cell glycans, thereby determining host range and tissue tropism.

**TABLE 2:** Functional Profiles of Influenza A Virus Proteins [27, 28, 30].

RNA segment	Encode proteins	Protein functionality
Segment 1	PB2	One of the RNA‐dependent RNA polymerase (RdRp) subunits, responsible for cap‐snatching‐i.e., recognition and cleavage of the 5’ cap‐containing host pre‐mRNAs—to prime viral mRNA synthesis.
Segment 2	PB1	A subunit of the RdRp complex that possesses both RNA chain‐elongation and endonuclease activities, catalyzing the processive elongation of nascent viral RNA strands.
Segment 2	PB1‐F2 (+1 ribosomal frameshift)	A small, 87‐amino‐acid pro‐apoptotic protein that potentiates viral pathogenicity by inducing mitochondrial dysfunction and subsequent apoptosis of host cells.
Segment 3	PA	A subunit of the RdRp complex exhibiting intrinsic proteolytic activity, which participates in both the synthesis and post‐transcriptional processing of viral RNA.
Segment 4	HA	A surface glycoprotein mediates receptor binding to host cells and subsequent membrane fusion, and constitutes the primary antigenic determinant.
Segment 5	NP	Associates with viral RNA to assemble the viral ribonucleoprotein complex (vRNP) and mediates nuclear import and packaging of the viral genome.
Segment 6	NA	A surface glycoprotein possessing sialidase (neuraminidase) activity that cleaves terminal sialic acid residues from host glycans, thereby facilitating virion release from infected cells.
Segment 7	M1, M2	Matrix protein 1 (M1) forms the inner layer beneath the viral envelope and orchestrates virion assembly and budding, whereas the M2 integral membrane protein functions as a proton‐selective ion channel. Upon endosomal entry, M2 equilibrates pH across the virion membrane, thereby facilitating uncoating of the viral nucleocapsid core.
Segment 8	NS1, NEP/NS2	Non‐structural protein 1 (NS1) functions as an interferon antagonist by suppressing interferon signaling pathways, thereby antagonizing the mammalian host's innate immune response. In contrast, nuclear export protein (NEP) facilitates CRM1‐dependent export of viral ribonucleoprotein complexes out of the nucleus into the cytoplasm, thereby facilitating progeny virion assembly.

In contrast, the stem region of HA undergoes a pH‐triggered conformational rearrangement upon acidification within the endosome, which catalyzes the merging between the viral and host endosomal membranes, resulting in the viral genome being released into the cytoplasm [28]. Moreover, evolutionary alterations in the receptor‐binding specificity of influenza virus hemagglutinin are crucial for cross‐species transmission. Understanding the patterns of antigenic variation is therefore essential for dissecting the molecular mechanisms that govern viral host‐range expansion and for enhancing influenza prevention and control strategies [28].

During replication, the RNA virus H5N1 exhibits an inadequate proofreading mechanism, rendering its polymerase susceptible to frequent base misincorporation. This phenomenon frequently results in the emergence of mutations (Table 3). These mutations not only modify the virus's receptor binding affinity but also significantly influence its infectivity, immune evasion capability, host range, and other characteristics.

**TABLE 3:** Impact of H5N1 Mutations on Viral Phenotype.

Mutation type	Mutation impact
T199I [35]	Enhances RBS flexibility, enabling engagement with diverse glycans.
V135M [31]	Located near the HA receptor site, this mutation may alter viral host preference.
T160A [36]	Potentially increasing affinity for human receptors.
R193N [17]	By altering RBS electrostatics, this mutation permits high‐affinity binding to human receptors.
Q226L [35]	It is associated with augmented complementarity with α‐2,6 sialic acid receptors.
N224K [43]	By altering electrostatics and the shape of the HA interface, it increases viral affinity for α‐2,6‐linked receptors.
E627K [41]	It increases viral RdRp activity in mammalian cells to drive efficient replication.
D701N [38]	Enhances viral replication via increased polymerase activity in mammalian cells.
H274Y [39]	By remodeling the NA active site, this mutation lowers inhibitor binding, leading to NAI resistance.
N294S [40]	By inducing a carbonyl flip at Tyr347, the mutation establishes a new hydrogen bond to Arg292, which sterically blocks the drug and lowers binding affinity.

HA of traditional H5N1 viruses primarily recognizes and binds to avian‐specific sialic acid receptors linked by α‐2,3 glycosidic bonds. This receptor selectivity facilitates efficient transmission among avian species, as the epithelial cell surfaces of their respiratory and digestive tracts are extensively covered with sialic acid (SA) receptors connected to galactose through α‐2,3 bonds (SAα‐2,3 Gal) [17]. In contrast, the epithelial cells on the surface of the human respiratory tract predominantly contain sialic acid (SA) receptors linked to galactose via α‐2,6 bonds (SAα‐2,6 Gal). The disparity in receptor specificity serves as the primary species barrier that hinders the transmission of the H5N1 virus from avian hosts to humans [31]. In recent years, emerging strains of H5N1 have undergone a series of critical mutations that have significantly enhanced their ability to bind to human‐type α‐2,6‐linked sialic acid receptors, thereby increasing their potential to infect human cells [32].

Among these mutations, the Q226L mutation is particularly critical as it facilitates a shift in the virus's receptor selectivity from avian‐type to human‐type receptors, thereby improving its complementarity with α‐2,6‐linked sialic acid receptors. Structurally, the glutamine residue at position 226, prior to mutation, specifically binds to α‐2,3‐linked sialic acid receptors through intermolecular forces such as hydrogen bonds. However, following the Q226L mutation, the glutamine residue is substituted with a leucine residue. The leucine side chain, being shorter and more hydrophobic, is less capable of forming effective hydrogen bonds with α‐2,3‐linked sialic acid receptors. Instead, this mutation demonstrates greater compatibility with the spatial configuration and chemical properties of α‐2,6‐linked sialic acid receptors, thereby enhancing its complementarity with these receptors [33].

For example, the T199I mutation identified in the H5N1 virus recently isolated from dairy cows has enhanced the virus's binding affinity specifically toward α‐2,3‐linked sialic acid glycans [17]. In the wild‐type virus, the threonine (T) residue at position 199, characterized by a short and highly hydrophilic side chain, restricts its binding affinity to α‐2,3‐linked sialic acid glycans. In the T199I mutation, threonine is substituted with an isoleucine (I) residue. Isoleucine, with its longer and more hydrophobic side chain, induces changes in the spatial conformation and chemical properties of the HA protein's binding pocket, thereby broadening its binding range to α‐2,3‐linked sialic acid glycans [34]. Consequently, the T199I substitution may influence the conformational flexibility of the receptor‐binding site (RBS), potentially enabling it to adopt a wider array of conformations and accommodate a more diverse spectrum of glycan moieties [35]. Additionally, the V135M mutation, which has a mutational prevalence of 15.4% within the B3.13 genotype, is frequently observed. This mutation, located in close proximity to the HA RBS, has been linked to modifications in viral receptor‐binding preferences, potentially influencing host adaptation [31]. The T160A substitution in the HA of bovine‐origin H5N1 eliminates the N‐linked glycosylation sequon at residue 158, a loss that may alter the virus's affinity for human‐type receptors [36]. Additionally, the R193N substitution is hypothesized to alter the electrostatic properties of the RBS, potentially facilitating steric configurations conducive to high‐affinity interactions with human‐type receptors [17].

The concurrent occurrence of the Q226L and N224K mutations collectively enhances the virus's binding affinity to α‐2,6‐linked sialic acid receptors. The N224K mutation involves the replacement of asparagine (N) with lysine (K), which has a longer side chain and a positive charge. This lysine residue interacts with the structural conformation induced by the Q226L mutation, further refining the spatial arrangement and electrostatic interactions at the binding interface between the HA protein and α‐2,6‐linked sialic acid receptors. These two mutations synergistically alter the overall charge distribution and spatial conformation of the receptor‐binding domain on the HA protein, thereby significantly enhancing the virus's affinity for α‐2,6‐linked sialic acid receptors [18].

However, this single substitution alone is insufficient to facilitate human‐to‐human transmission. Instead, the synergistic effects of additional mutations, such as the E627K substitution in polymerase basic protein 2 (PB2) and the N224K substitution in NP, are necessary to enhance viral infectivity and replicative fitness within the human host [37], thereby broadening the transmission potential. This enhancement suggests the possibility for the virus to overcome the species barrier, facilitating transmission from avian hosts to mammals, including humans.

The H5N1 virus has undergone evolutionary changes that enhance its pandemic potential through a series of mutations across various functional proteins, each contributing uniquely to host adaptation. Firstly, mutations in the HA protein, specifically Q226L and G228S, serve as pivotal determinants of receptor specificity. Alongside T199I, these alterations significantly increase the virus's affinity for human‐type receptors, thereby directly enhancing cross‐species transmissibility [35, 36, 38, 39, 40]. Secondly, adaptive mutations in the PB2 polymerase subunit, particularly E627K and D701N, are critical markers of polymerase adaptation. These mutations significantly enhance the enzymatic activity of the viral RdRp in mammalian cells, promoting robust viral replication, increased pathogenicity (including neuroinvasiveness), and rapid selective fixation, all of which facilitate viral adaptation to mammalian hosts [38, 41]. Lastly, distinct from the aforementioned adaptation markers, mutations in the NA protein, such as H274Y and N294S, primarily confer drug resistance. Although these mutations significantly decrease susceptibility to neuraminidase inhibitors (NAIs) such as oseltamivir—evidenced by the H274Y mutation increasing the IC_50_ by several hundred‐fold [39], they may entail trade‐offs in viral fitness. Nonetheless, specific variants, such as N294S, can arise and persist even without antiviral pressure, while maintaining transmissibility [40]. This persistence considerably limits therapeutic options.

Moreover, substitutions in the H5N1 lead to changes in the structure of its surface antigens. In the non‐mutated H5N1 virus, the antigenic epitopes of the HA protein possess specific amino acid sequences and spatial conformations that are recognizable by the immune system of hosts previously vaccinated against or infected with the virus. However, the occurrence of mutations, such as amino acid substitutions at critical sites, leads to changes in the amino acid sequences and spatial conformations of the antigenic epitopes on the HA protein. For example, the Q226L and N224K mutations significantly impact the spatial structure of the antigenic epitopes on the globular head of the HA protein [18]. Consequently, antibodies derived from hosts that have been vaccinated or infected are unable to effectively recognize and bind to the mutated H5N1 virus, enabling the virus to evade the host's immune defense [15]. As mutations continue to accumulate, the risk of infection with newly mutated strains increases for populations that previously possessed a certain level of immunity against the H5N1 virus.

The H5N1 virus evades humoral immunity through antigenic drift in the HA head domain, giving rise to novel clades every 2–10 years [42]. Original antigenic sin imprints a bias toward cross‐reactive, low‐avidity B‐cell responses; although subsequent affinity maturation effectively neutralizes escape variants of the priming strain, this maturation process simultaneously widens the antigenic distance at which drifted variants can evade neutralization [42]. This apparent paradox originates from the epistatic network within HA: whereas mature antibodies elicit extensive escape profiles against drifted strains (e.g., 34 distinct sites for SI06), no such escape is observed against the ancestral strain (SN89), indicating that the epistatic architecture of HA expands the mutational routes available for immune evasion [42].

## 3 Prospects for Future Control and Prevention of H5N1 Epidemics

**FIGURE 3:**
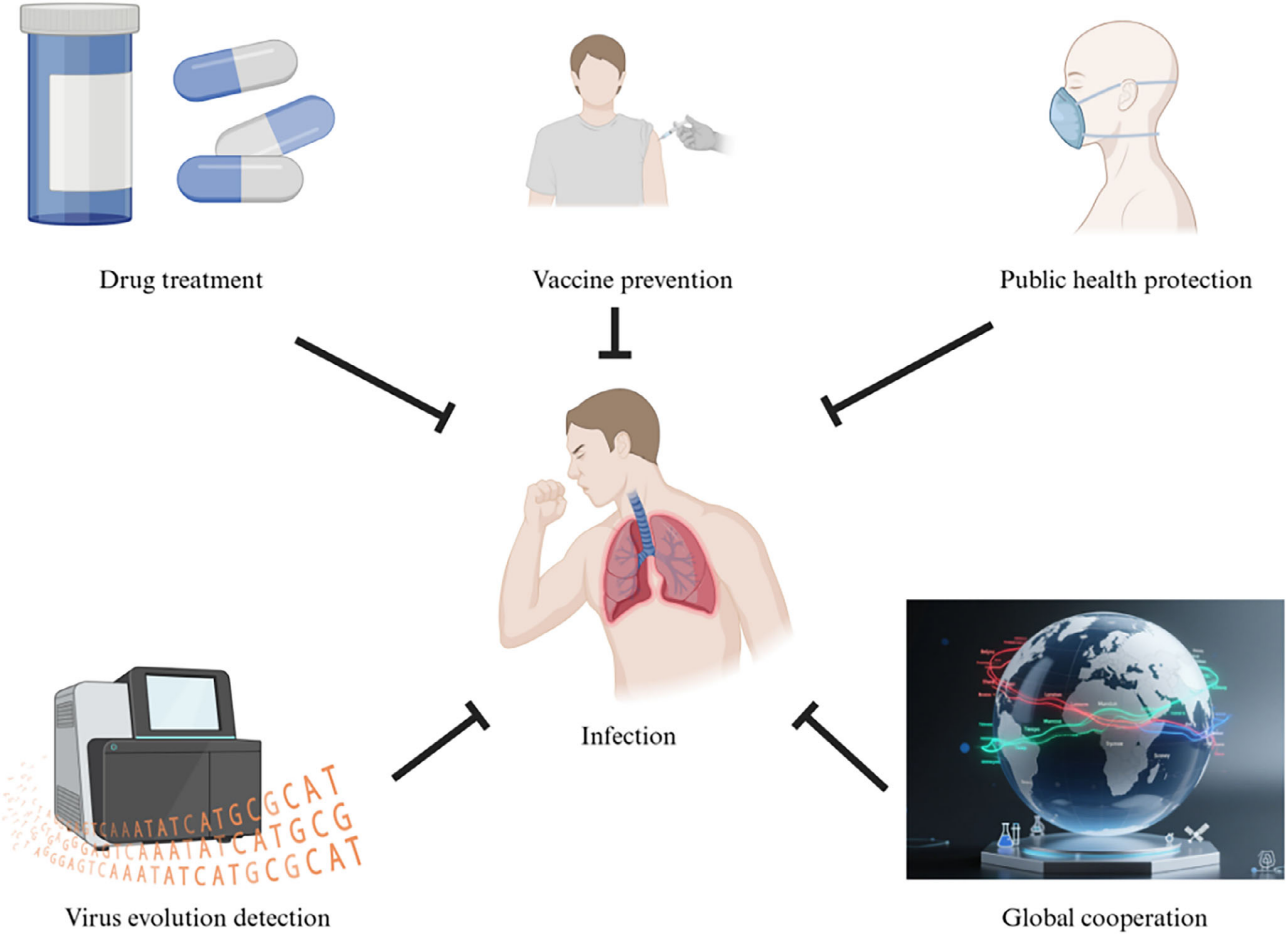
Schematic diagram of prevention and control measures. This diagram illustrates the multi‐dimensional strategies against H5N1 infection, including drug treatment, vaccine prevention, public health protection, virus evolution detection, and global cooperation. All these strategies focus on the core process of preventing and protecting against the occurrence of H5N1 infection.

Against the backdrop of global efforts to address the current epidemic and prevent potential future outbreaks, adopting preventive measures against the H5N1 virus has become increasingly crucial (Figure 3) [44]. Infected avian species can shed the H5N1 virus through saliva, mucus, and feces, posing a risk of transmission to humans through contact with such birds. Consequently, the most effective strategy for preventing H5N1 infection is to minimize exposure to potential viral sources as much as possible [45]. It is important to note that the majority of human H5N1 cases arise following close contact with infected birds, with the risk of transmission further heightened when individuals touch their mouth, eyes, or nose after such contact [46, 47]. Public awareness of H5N1 is crucial, and it is essential to emphasize avoiding of contact with dead or sick birds and mammals, as well as those behaving abnormally, unless individuals have received professional training and are equipped with appropriate protective gear. Additionally, it is necessary to prevent pets, like cats, from interacting with potentially infected animals. These preventive measures are not only relevant in rural settings but also critical in urban areas, including parks and green spaces where wild bird populations are concentrated, as the risk of exposure in these areas is relatively higher [48].

If the virus is permitted to accumulate mutations that enhance its adaptation to mammals, particularly through reassortment with seasonal human influenza viruses, the risk of the 2.3.4.4b clade potentially instigating the next pandemic is progressively increasing, although this risk is not precisely quantifiable [49]. Despite the challenges in quantifying the exact pandemic risk, it is imperative to urgently implement a series of measures aimed at mitigating the overall risk [25]. By optimizing epidemiological investigations, enhancing biosecurity protocols, and promoting vaccination to decrease viral prevalence in key agricultural species, the likelihood of zoonotic transmission can be reduced [44]. Prevention and mitigation strategies should encompass educating and training high‐risk groups, supplying appropriate personal protective equipment, and administering H5N1 vaccines to decrease the incidence of H5N1 infection in humans. Additionally, public education initiatives aimed at raising awareness about the dangers of H5N1 and the potential risks associated with contact with wild animals, even in urban environments, can further diminish the likelihood of infection. Furthermore, vaccinating high‐risk populations with seasonal influenza vaccines to mitigate their likelihood of contracting seasonal human influenza can reduce the incidence of co‐infections, thereby diminishing the risk of viral reassortment [25, 44, 49, 50].

Antiviral agents and vaccines continue to be the primary strategies for controlling H5N1; current therapeutic approaches predominantly target the NA, HA, RdRp, NP, and the M2 ion channel protein [50, 51].

NAIs function by competitively inhibiting NA enzymatic activity, thus preventing viral release and dissemination [50, 51]. Several NAIs are currently approved for the treatment of H5N1 infection, including orally administered oseltamivir, whose active carboxylate metabolite competitively occupies the NA active site, thereby inhibiting the detachment and spread of nascent virions from the host‐cell membrane [51]. This mechanism remains highly effective against P194S mutant strains [52]. The inhaled dry‐powder formulation of zanamivir establishes multiple hydrogen bonds within the NA active site, competitively inhibiting sialic‐acid cleavage and thereby blocking virion budding [53]. Intravenously administered peramivir adopts a distinctive binding mode within the NA active site, retaining partial affinity even in the presence of canonical resistance substitutions at E119, R292, and H274; consequently, it retains efficacy against certain oseltamivir‐resistant strains [52].

HA inhibitors impede viral entry by competitively occupying a conserved pocket [51, 54] in the HA head region—comprising the 190‐helix, 130‐loop, and 220‐loop—with sialic acid (Neu5Ac) derivatives that mimic the natural receptor; however, structural homology limits their intrinsic binding affinity [54]. Tert‐butylhydroquinone (TBHQ) associates with the prefusion HA trimer at the inter‐subunit interface, stabilizing the neutral‐pH conformation and thereby preventing the low‐pH‐triggered conformational rearrangements requisite for viral–endosomal membrane fusion [55].

RdRp‐targeting inhibitors are categorized into complex‐assembly blockers, PB2 cap‐binding antagonists, PA endonuclease inhibitors, and nucleos(t)ide analogues [50]. Among these agents, the purine nucleoside analogue favipiravir (Avigan) is integrated into newly synthesized viral RNA, leading to the accumulation of transition mutations that result in lethal mutagenesis and subsequently inhibit viral replication. It has exhibited significant antiviral efficacy against H5N1 viruses [50].

NP, a central element of the viral ribonucleoprotein complex (vRNP), orchestrates viral RNA replication and transcription [50]. The small‐molecule inhibitor FA‐6005 traps NP in the I41 pocket, thereby blocking vRNP nuclear export and abrogating viral RNA transcription/replication (EC_50_ = 1.6–6.2 µm [56]). Additionally, it hinders virion budding. Its mechanism of action is mechanistically distinct from that of NAIs or PA inhibitors, conferring substantial potential for combination regimens.

The M2 ion channel protein assembles into a homotetrameric transmembrane pore that prevents premature HA conformational changes [57] and additionally orchestrates virion uncoating and maturation [50]. The M2 inhibitor 1‐isocyanoadamantane exhibits ten‐fold higher potency against wild‐type H5N1 M2 compared with amantadine (EC_50_ = 0.49 µm), yet it is inactive against S31N or V27A variants [58]. Conversely, 3‐azatetracyclanes retain efficacy against both wild‐type and V27A mutant M2, and oral administration of this compound at a dosage of 10 mg kg^−^^1^ in murine models significantly reduces pulmonary viral titers [59].

Substitutions in the surface proteins of H5N1 not only enhance the virus's ability to cross species barriers but also reduce the binding affinity between drugs and their targets, thereby leading to drug resistance [60]. Therefore, developing broad‐spectrum antiviral drugs is of great significance. Broad‐spectrum antiviral drugs can act by targeting relatively stable conserved sites—which are less prone to mutations— in the viral life cycle; they exhibit greater tolerance to viral drug‐resistant mutations. These drugs not only inhibit viral replication but also enhance the host's immune response, providing a more effective solution for addressing the H5N1 virus and other emerging viruses.

Vaccination represents the paramount and most efficacious strategy for the prevention and management of influenza virus infections and associated illnesses. The United States Food and Drug Administration (FDA) has granted approval for three H5N1 vaccines, developed by Sanofi‐Pasteur, CSL Seqirus, and GlaxoSmithKline (ID Biomedical Corporation of Quebec) [61]. These vaccines are specifically designed to target strains that were prevalent in the early 2000s, namely A/Vietnam/1194/2004, clade 1, and A/Indonesia/5/2005, clade 2.1 [61]. Although studies have assessed the binding, hemagglutination inhibition, and neutralizing antibody responses elicited by three approved vaccines in adults, demonstrating their capacity to induce cross‐neutralizing antibodies against highly pathogenic H5N1 clade 2.3.4.4b influenza viruses and indicating their potential as interim vaccines, it remains crucial to expedite the development of vaccines specifically targeting this clade [61].

The United States Centers for Disease Control and Prevention (CDC) and the World Health Organization (WHO) Collaborating Centers have developed several candidate vaccine viruses (CVVs) specifically targeting this clade [62]. Vaccine manufacturers have utilized these CVVs to produce vaccines that comply with Good Manufacturing Practice standards, and clinical trials are currently in progress. However, the rapid and large‐scale production of these vaccines during a highly pathogenic avian influenza (HPAI) pandemic may encounter significant challenges [63]. Consequently, researchers are exploring alternative vaccine strategies for the 2.3.4.4b A (H5) group. These strategies aim to eliminate the necessity for large‐scale viral cultivation, thereby enabling the flexible and timely iteration of vaccine strains to keep pace with the virus's rapid evolution.

On the one hand, the significant success of mRNA vaccines in addressing the COVID‐19 pandemic has catalyzed their investigation for application against other infectious diseases, such as influenza [64]. In a rapid preclinical assessment, Hatta et al. [63]. Evaluated an mRNA vaccine targeting highly pathogenic avian influenza (HPAI). This vaccine, encapsulated within lipid nanoparticles (LNPs) and directed against the 2.3.4.4b H5 virus clade, was demonstrated to elicit robust neutralizing antibody responses and confer protection to ferrets against lethal H5N1 viral challenges. Furthermore, sera from vaccinated ferrets effectively neutralized a human‐acquired HPAI A (H5N1) isolate, which was closely linked to the 2024 outbreak in dairy cattle in Texas, USA. These findings underscore the efficacy of the mRNA vaccine against HPAI A (H5N1) and underscore its potential as a platform for future pandemic influenza vaccines.

In the pursuit of optimizing mRNA vaccine platforms, recent research has concentrated on enhancing safety profiles. Kawai et al. [65] have developed an innovative liposome‐based delivery system, termed ssPalmO, which incorporates disulfide bonds to facilitate self‐degradation. This novel mRNA‐LNP formulation not only elicited superior Th1 immune responses and provided cross‐protection against heterologous H5N1 virus in murine models, as compared to conventional LNPs, but also significantly mitigated adverse events such as systemic inflammation and fever, owing to its rapid degradation in vivo. This approach offers a promising strategy for the development of highly effective and low‐toxicity H5N1 mRNA vaccines [65].

On the other hand, influenza virus‐like particle (VLP) vaccines elicit broad immune responses and offer cross‐protection against various influenza strains through different vaccination routes, making them a promising alternative to traditional inactivated vaccines [66, 67]. Recent studies indicate that a single dose of the H5N1 VLP vaccine can generate high‐titer HI antibodies and cross‐protect against multiple subtypes, with effectiveness significantly affected by the vaccine dose and choice of adjuvant [68].

Furthermore, the development of multivalent vaccines, which are capable of targeting multiple subtypes or variants of the H5N1 virus simultaneously, represents an effective strategy for addressing viral diversity and mutations. A recent study assessed the efficacy of a trivalent inactivated vaccine comprising strains of H5N1 (clades 2.2.1.1 and 2.2.1.2) and H5N8 (clade 2.3.4.4b) against various clades of highly pathogenic avian influenza (HPAI) viruses [69]. The study's core findings indicated that the vaccine conferred robust and broad‐spectrum protection, achieving 100% survival against H5N1 challenges and significantly reducing the risk of viral transmission [69].

At the same time, an optimized clade 2.3.4.4b H5N1 inactivated vaccine, administered intranasally, effectively induces strong mucosal and systemic immunity in mice, offering full protection against different virus strains [70]. Nonhuman primate studies highlight the importance of mucosal immunity, showing that adenovirus‐vectored and mRNA‐based H5 vaccines provide protection, with an intratracheal booster after an intramuscular prime yielding enhanced immunity [71] (Table 4).

**TABLE 4:** Pharmacotherapy for H5N1 Influenza.

Drug class	Exemplar	Target/Mechanism of action	Activity/Advantage against H5N1
NAIs	Oseltamivir Zanamivir Peramivir [52, 53, 72]	Inhibits NA activity, thereby precluding virion release from infected cells [50, 51].	Exhibits subnanomolar potency against both wild‐type and drug‐resistant viral strains. [50, 51]
HA inhibitors	Sialic acid derivatives (Neu5Ac) TBHQ [54, 55]	Blocks HA‐mediated viral attachment to host cells or subsequent membrane fusion. [51]	Targets conserved epitopes within the HA globular domain; several agents exhibit direct, sub‐micromolar inhibitory activity against H5N1. [51]
RdRp inhibitors	Favipiravi(Avigans) [50]	Suppresses the viral RdRp complex, thereby arresting viral RNA replication and transcription. [50]	Targets the highly conserved RdRp complex, conferring broad‐spectrum antiviral activity. [50]
NP inhibitors	FA‐6005 [56]	Disrupts NP function, thereby preventing vRNP assembly or nuclear export. [56]	It targets conserved, function‐critical regions of NP, and several lead compounds have demonstrated the capacity to potentiate existing therapeutic regimens. [50, 51]
M2 ion‐channel protein inhibitors	1‐isocyanoadamantane, 3‐azatetracyclanes [58, 59]	Blocks the M2 ion channel, thereby inhibiting virion uncoating and maturation. [50, 59]	Targeting the M2 channel, certain derivatives can circumvent established resistance mutations, yet their overall antiviral potency remains inferior to that of other drug classes. [50, 51, 58, 59]

**FIGURE 4:**
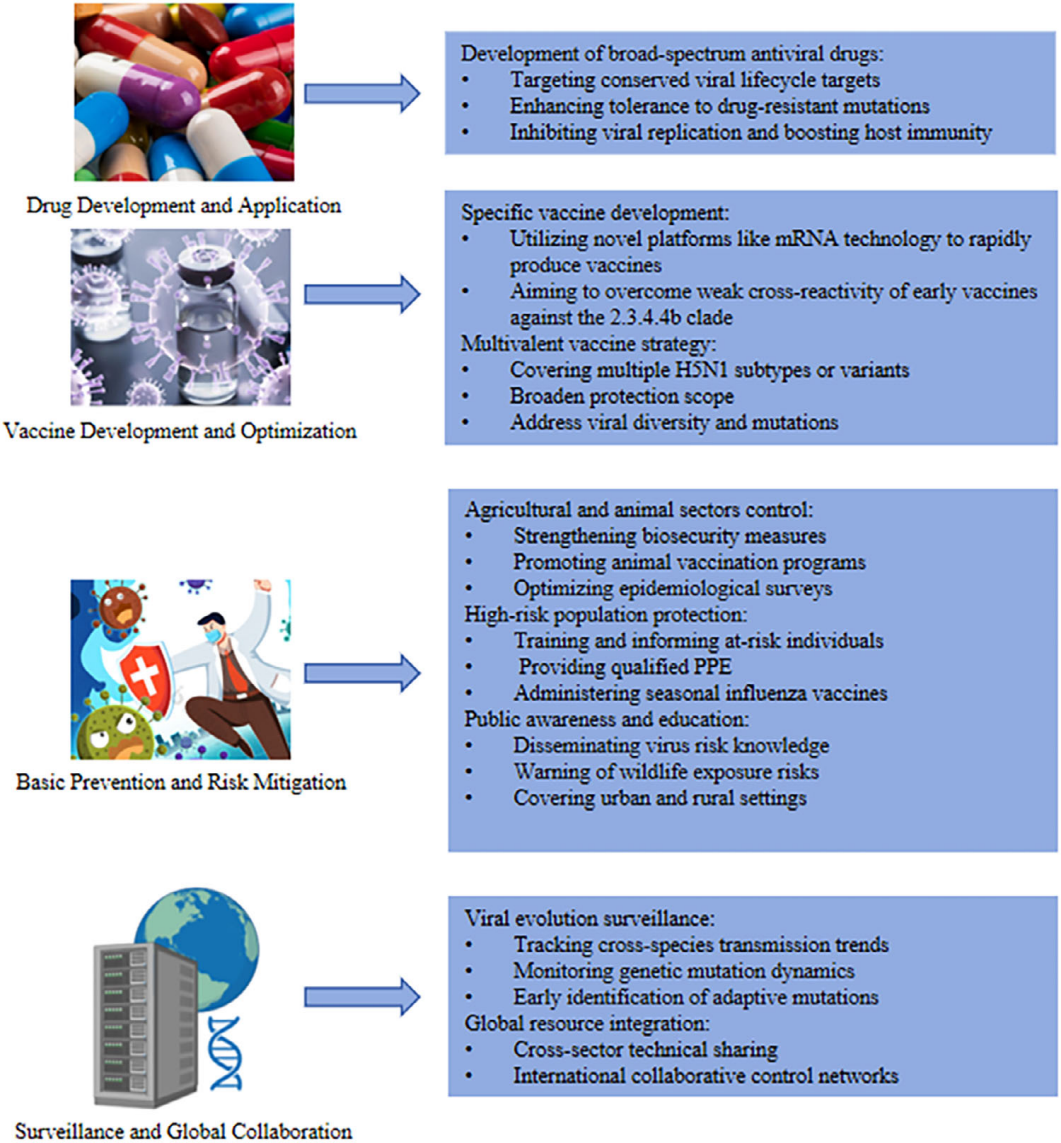
Prevention and Control of H5N1 Virus Transmission in Humans and Animals. This figure presents a comprehensive strategy for preventing and controlling H5N1 virus transmission, covering four major aspects as follows: drug research and development application, vaccine research and development optimization, basic prevention and control with risk reduction, and surveillance and global collaboration.

Although the current transmission efficiency of the H5N1 virus among humans remains limited, its highly variable genome provides the molecular foundation for potential evolution toward sustained human‐to‐human transmission [73]. Significant knowledge gaps remain concerning the key factors that enable efficient human‐to‐human spread of the virus, its long‐term evolutionary trajectory in new mammalian hosts such as cattle, and the impact of continuous surface antigen drift on the efficacy of antibody protection [74, 75, 76]. To address these issues, future global surveillance efforts must extend beyond poultry populations to systematically encompass domestic and wild mammalian species, particularly those in outbreak areas, in order to establish a comprehensive ‘One Health’ early warning system [77]. Addressing this threat necessitates global collaboration in the integration of resources and technologies to continuously monitor viral evolutionary dynamics, with a particular emphasis on its cross‐species transmission tendencies from poultry to humans and other mammals. By implementing comprehensive strategies that encompass surveillance, research, and international cooperation (Figure 4), we can more effectively mitigate future H5N1 virus threats, reduce the risk of a global pandemic, and safeguard both human health and ecological balance.

## Author Contributions

Conceptualization was conducted by H.F., Z.X., H.F., B.H., and H.Z.; funding acquisition was carried out by H.F.; validation was performed by W.M., L.D., and Y.L.; supervision was provided by H.F., Z.X., H.F., B.H., and H.Z.; visualization was performed by L.D.; writing – original draft was prepared by W.M., L.D., and Y.L.; writing – review and editing were performed by W.M., L.D., Y.L., B.H., H.Z., and H.F. All authors read and agreed to the published version of the manuscript.

## Conflicts of Interest

The authors declare no conflicts of interest.

## Data Availability

The data that support the findings of this study are available in the supplementary material of this article.
